# An in vitro study on dentin abrasion comparing two sonic toothbrushes with and without a coating versus an ADA reference manual toothbrush

**DOI:** 10.1371/journal.pone.0333705

**Published:** 2025-10-07

**Authors:** Lea Walter, Amir Aminy, Stefan Zimmer, Mozhgan Bizhang

**Affiliations:** Department of Operative and Preventive Dentistry, Witten/Herdecke University, Witten, Germany; Danube Private University, AUSTRIA

## Abstract

This in vitro study evaluated dentin abrasion and surface roughness caused by two sonic toothbrushes with different filament coatings compared with those caused by a standard ADA reference manual toothbrush. Standardized dentin samples (n = 8 per group) were prepared from bovine incisors, embedded in acrylic blocks, polished, and hardness-verified using the Vickers method. A central brushing area was created by masking two reference zones with adhesive tape. Brushing simulations were performed in a laboratory device for 10,000 cycles at a load of 1.5 N using a toothpaste slurry (RDA 129) that was prepared at a 1:1.6 water-to-paste ratio. The tested toothbrushes included the ADA reference manual toothbrush (uncoated nylon filaments), the Curaprox Hydrosonic Pro toothbrush (uncoated PBT filaments), and the Curaprox Hydrosonic Black is White toothbrush (charcoal-coated PBT filaments). Dentin loss was measured by noncontact optical profilometry, and surface roughness was assessed by determining the Ra, Rq and Sa values. Data normality was verified using the Shapiro–Wilk test, and differences among groups were analyzed by one-way ANOVA with Bonferroni correction (α = 0.05). Compared with the ADA reference manual toothbrush, both the sonic toothbrushes caused significantly greater dentin loss (p < 0.001), with no significant difference between the sonic models (p > 0.05). The changes in roughness followed a similar pattern. These findings suggest that, under standardized laboratory conditions, sonic toothbrushes, regardless of the use of a filament coating, cause more dentin wear than a manual reference brush. Moreover, greater dentin abrasion was associated with less roughness, indicating an inverse relationship between tissue loss and surface roughness.

## Introduction

Toothbrushing is the most widely used method for mechanically removing plaque and preventing caries and periodontal disease [1–4]. While both manual and electric toothbrushes are effective for plaque control, variations in brush head design, filament material, filament diameter, tuft density, and filament coating can influence their abrasivity [5,6]. Among dental hard tissues, dentin is particularly vulnerable to mechanical wear because of its lower hardness (≈66 HV) than that of enamel (≈275 HV) [7]. Abrasion of dentin is most common in exposed cervical areas, where enamel is thin and dentin is rapidly exposed [8].

Electric toothbrushes, particularly sonic models, have gained popularity because of their high-frequency bristle motion and potential for achieving superior plaque removal [9]. In addition to differences in filament geometry and arrangement, manufacturers have introduced surface modifications, such as activated charcoal coatings, to enhance tooth whitening [10]. Charcoal particles are marketed for their potential stain-removal effect; however, while their use and abrasivity in toothpaste formulations have been widely studied [11–14], the potential effects of charcoal-coated toothbrush filaments on dentin wear and surface texture have, to the best of our knowledge, not yet been thoroughly investigated under standardized laboratory conditions.

The abrasivity of toothbrushes can be measured using methods described in ISO 11609, including the radioactive concentration method of the American Dental Association (ADA) and surface profilometry [15]. Noncontact optical profilometry provides high precision and reproducibility, enabling the quantification of both vertical dentin loss and surface roughness without requiring physical contact with the sample surface [16]. Previous in vitro work by Bizhang et al. [17] also used the ADA reference manual toothbrush as a control, facilitating comparability across abrasion studies. These authors reported that, under standardized conditions, electric toothbrushes caused significantly greater dentin abrasion than the ADA manual reference toothbrush. Therefore, the same ADA reference model was used in the present study to ensure methodological comparability with previous work and to allow our findings to be placed in the context of established evidence on toothbrush abrasivity.

Therefore, the aim of this in vitro study was to compare dentin abrasion and surface roughness caused by two sonic toothbrushes—one with charcoal-coated filaments (Curaprox Hydrosonic Black is White) and one with uncoated filaments (Curaprox Hydrosonic Pro)—to those caused by an ADA reference manual toothbrush under standardized brushing conditions. The specific objectives were (1) to quantify vertical dentin loss using noncontact profilometry and determine whether a charcoal filament coating influences abrasion and (2) to evaluate postbrushing surface roughness (Ra, Rq and Sa) among all the tested toothbrushes. Based on these objectives, the following null hypotheses (H₀) were formulated:

- H01: There is no statistically significant difference in dentin abrasion caused by the charcoal-coated sonic toothbrush, the uncoated sonic toothbrush, and the ADA reference manual toothbrush.
- H02: There is no statistically significant difference in surface roughness caused by the charcoal-coated sonic toothbrush, the uncoated sonic toothbrush, and the ADA reference manual toothbrush.

## Materials and methods

This study was conducted entirely in vitro and did not involve any human participants, identifiable human data, or experimentation on live animals. The bovine teeth that were used in this research were obtained from a commercial supplier and were not specifically collected for this study. Therefore, ethical approval was not needed.

Bovine dentin was selected because of its structural similarity to human dentin and its widespread acceptance as a substitute material in abrasion studies. This choice is consistent with the recommendations of ISO 11609, which recognizes bovine dentin as a suitable substrate for standardized brushing simulations [15].

### Sample size calculation

Sample size calculation was performed using G*Power (Version 3.1, University of Düsseldorf, Germany) [18] for one-way ANOVA (fixed effects, omnibus) with three independent groups. The effect size was derived from dentin abrasion data that were reported in a previous study with a comparable experimental setup [17]. With α = 0.05, statistical power (1–β) = 0.80, and equal group sizes, the analysis indicated a minimum of three specimens per group. To account for potential dropouts and to ensure the robustness of the statistical analysis, the final sample size was set to eight specimens per group.

### Specimen preparation

Standardized dentin samples were prepared from bovine incisors that were obtained from a commercial supplier (Libreco Food Service, Gelsenkirchen, Germany). Cylindrical samples (5 mm diameter) were harvested from the buccal cervical region using a trephine bur (Hager & Meisinger, Neuss, Germany) under constant water cooling. Enamel was removed using 500-grit abrasive paper.

The dentin cylinders were embedded in clear prosthetic resin (Palapress, Heraeus Kulzer, Hanau, Germany) and polymerized under 2 bar of pressure for 20 minutes. The sample surfaces were then polished sequentially using 500-, 1000-, 2500-, and 4000-grit abrasive papers on a precision grinding system (Exakt 400/40, Exakt Technologies, Norderstedt, Germany) until a surface roughness below 0.1 µm was achieved. This was verified using noncontact optical profilometry (InfiniteFocus G3; Alicona, Graz, Austria).

To ensure material homogeneity, all the samples were subjected to Vickers microhardness testing prior to brushing. Indentations were placed outside the brushing zone, as recommended by ISO 11609. Measurements were performed using a microhardness tester (Wilson VH1202, Buehler, Illinois, USA) with a 3 N load applied for 15 seconds. Only specimens with hardness values between 30 and 70 HV were included in accordance with the ISO standard [15]. The hardness values of the selected samples ranged from 52.37 to 59.77 HV (minimum to maximum).

To measure abrasion, the dentin surface was partially masked using two lateral strips of adhesive tape (Tesa 60454; Beiersdorf, Hamburg, Germany), creating two unbrushed reference areas and one central brushing zone. These reference areas remained protected during the brushing protocol and served as baselines for vertical substance loss measurement.

### Toothbrushes

Three toothbrush types were tested: the ADA reference manual toothbrush, an uncoated sonic toothbrush (Curaprox Hydrosonic Pro), and a charcoal-coated sonic toothbrush (Curaprox Hydrosonic Black is White). Before testing, all the toothbrushes were soaked in distilled water for 24 hours in accordance with ISO 11609 [15]. Except for the charcoal coating, no other differences in brush head design or filament characteristics were observed between the two sonic toothbrush models. A detailed overview of the design of the tested toothbrushes is provided in Table 1.

**Table 1:** Specifications of the Toothbrushes and their Bristles.

Design of toothbrush	Toothbrush	Filament material	Coating	Filament diameter (mm)	Number of tufts	Filaments per tuft	Total filaments
**Manual Flat-trim**	ADA reference manual toothbrush	Nylon	None	0.18	47	30	1,410
**Sonic**	Curaprox Hydrosonic pro	PBT (polybutylene terephthalate)	None	0.15	26	62	1,612
**Sonic**	Curaprox Hydrosonic Black is White	PBT (polybutylene terephthalate)	charcoal-coated	0.15	26	62	1,612

Design characteristics of each toothbrush, including the filament material, coating, filament diameter, number of tufts, number of filaments per tuft, and total filament count. The data are based on the manufacturer’s specifications.

### Slurry preparation

A toothpaste slurry was prepared using Dentalux Seiden Weiß (Lidl), a commercially available toothpaste with a reported RDA value of 130 (manufacturer’s information). The toothpaste was mixed with distilled water at a 1:1.6 ratio by weight and homogenized using a magnetic stirrer, in accordance with ISO 11609 [15].

### Toothbrushing machine and brushing protocol

**Fig 1:**
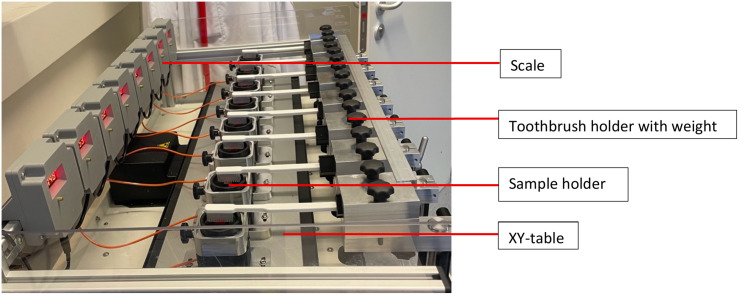
Toothbrushing Machine (ZM-3; Mechatronik, Feldkirchen-Westerham, Germany).

All the samples were brushed using a standardized brushing simulator (ZM-3; Mechatronik, Feldkirchen-Westerham, Germany) (Fig 1). The samples were mounted in custom 3D-printed holders and subjected to linear brushing movements (stroke length: 10 mm; speed: 20 mm/s) under a constant load of 1.5 N. Eight specimens were brushed simultaneously to ensure uniform experimental conditions.

To accommodate the ADA reference manual toothbrush, a customized 3D-printed holder was designed and fabricated to ensure stable positioning within the simulator. For the sonic toothbrushes, a different mounting approach was used: the broader handle dimensions allowed secure fixation directly in the simulator’s standard clamp. In all cases, brush head alignment relative to the sample surface was verified before each run to ensure consistent angulation, full bristle contact, and uniform pressure application.

Brushing parameters were based on ISO 11609 [15], which specifies 4,000 initial cycles followed by an additional 6,000 cycles for the reference toothbrush. In this study, the total number of brushing cycles was maintained at 10,000, but a modified sequence of 8,000 initial cycles and 2,000 subsequent cycles was applied to ensure measurable and consistent abrasion. Sonic toothbrushes (Hydrosonic Pro and Hydrosonic Black is White, Curaden, Stutensee, Germany) were operated in the standard mode described by the manufacturer, that is, at 64,000 strokes/min, throughout the procedure.

Brushing duration was designed to simulate long-term clinical use. Assuming two 2-minute brushing sessions per day, 10,000 cycles correspond to approximately six weeks of use in the same area. For isolated specimens, this equates to approximately eight years of simulated use, based on the approximation that 5 seconds of brushing represents one full day of oral hygiene [8].

To confirm procedural validity, the proportional linearity of dentin loss was assessed in accordance with ISO 11609 [15]. The ratio of mean abrasion depths after 10,000 and 8,000 cycles with the ADA reference manual toothbrush yielded a linearity factor of 1.23, which is within the ISO-defined acceptable range of 1.1–1.4.

### Measurement of dentin abrasion

Dentin abrasion was quantified using noncontact optical profilometry (InfiniteFocus system). Three-dimensional panoramic images were captured at 10 × magnification and were composed of six stitched individual images per sample. Before imaging, the specimens were thoroughly cleaned and dried. Autofocus and lighting were adjusted to capture both brushed and unbrushed areas within the same depth range.

**Fig 2:**
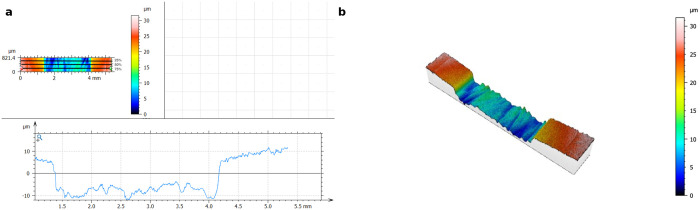
Representative Illustration of a Brushed Dentin Specimen Analyzed in Mountains 8 Software. **(a)** Two-dimensional color map of a brushed dentin specimen with the corresponding profile line. **(b)** Three-dimensional surface reconstruction of the same specimen illustrating the vertical substance loss. The color scale indicates depth values in micrometers (µm).

The panoramic images were exported and analyzed using Mountains 8 software (Digital Surf, Besançon, France). Each 3D image was converted into a two-dimensional projection (Fig 2), and three linear profile measurements were taken per sample at standardized positions (25%, 50%, and 75% of the sample width). These locations are visually indicated in the figure to clarify the measurement sites. The differences in vertical height between the central brushed area and adjacent unbrushed reference zones were determined using the software’s area measurement function. The mean of the three measurements was calculated to determine the average abrasion depth for each sample.

To compare the relative abrasivity of different toothbrush types, a profilometry**-**equivalent RDA (RDA-PE) value was calculated by setting the vertical dentin loss measured for the ADA reference manual toothbrush to 100. The profilometry-equivalent RDA (RDA-PE) values for the two sonic toothbrushes were obtained by normalizing their mean abrasion depths to this reference value.

### Surface roughness analysis

Surface roughness was evaluated using the same optical profilometry system (InfiniteFocus system) following the brushing procedure. The following roughness parameters were recorded: arithmetical mean roughness (Ra), root mean square roughness (Rq) and arithmetical mean height (areal surface roughness (Sa) were recorded. Measurements were taken at the center of each brushed area using a standardized vertical line scan.

To ensure comparability, the cutoff wavelength (λc) for roughness measurement was set to 25 µm. Three scans were performed per sample, and the mean values were calculated for statistical analysis. All individual roughness measurements are provided in the Supporting Information (S1 and S2 Files).

### Statistical analysis

All the statistical analyses were performed using IBM SPSS Statistics, version 23 (IBM, Armonk, NY, USA). The normal distribution of the data was assessed using the Kolmogorov‒Smirnov and Shapiro‒Wilk tests [19]. Parametric tests were applied as all the datasets met the assumption of normality (p > 0.05) [20]. Group differences in dentin abrasion and surface roughness were analyzed using one-way analysis of variance (ANOVA) [21], followed by Bonferroni post hoc correction for multiple comparisons [22]. Statistical significance was defined as p < 0.05 [23]. All the data are presented as the mean values ± standard deviations, with corresponding 95% confidence intervals.

## Results

The normal distribution of the dentin loss and surface roughness data in all the groups was confirmed by the Kolmogorov–Smirnov and Shapiro–Wilk tests (all p > 0.05). Statistically significant differences in both dentin loss and surface roughness were observed between the toothbrush groups (one-way ANOVA, p < 0.001).

The greatest amount of dentin loss was observed after brushing with the Curaprox Hydrosonic Pro, followed by Curaprox Hydrosonic Black is White. The ADA reference manual toothbrush caused the least amount of abrasion. The abrasion caused by both the sonic toothbrushes was significantly greater than that caused by the ADA reference manual toothbrush (p < 0.001), whereas no significant difference was detected between the two sonic brushes (p > 0.05). For comparative purposes, the profilometry equivalent RDA value for the ADA reference manual toothbrush was set to 100. The RDA-PE values for Curaprox Hydrosonic Pro and Curaprox Hydrosonic Black is White, which were calculated by normalizing their mean abrasion depths to the ADA reference and were 173.61 and 147.72, respectively.

Surface roughness was highest after brushing with the ADA reference manual toothbrush and lowest after brushing with the Curaprox Hydrosonic Pro. Both types of sonic toothbrushes produced dentin surfaces with significantly less roughness than the manual toothbrush (p < 0.001), with no significant differences between them. All the results are summarized in Tables 2 and 3.

**Table 2:** Mean and Standard Deviation (SD) of Vertical Dentin Loss (µm) and Profilometry-Equivalent RDA (RDA-PE) Values Relative to the ADA Reference Manual Toothbrush.

Toothbrush	Mean (μm)	Standard deviation (μm)	95% Confidence Interval (μm)	Profilometry equivalent RDA-PE
**ADA reference manual toothbrush**	13.97^a^	± 2.74	10.85–19.17	100^a^
**Curaprox Hydrosonic Pro**	23.46^b^	± 1.49	21.23–25.73	173.61^b^
**Curaprox Hydrosonic Black is White**	20.11^b^	± 1.01	18.86–21.49	147.72^b^

Within each column, values sharing the same superscript letter are not significantly different (p > 0.05), whereas values with different letters differ significantly (p < 0.001; one-way ANOVA with the Bonferroni post hoc correction).

**Table 3:** Mean Surface Roughness (Ra, Rq, and Sa; μm) after Brushing with Different Toothbrushes. The values are presented as the mean ± standard deviation (SD) and 95% confidence interval (CI).

Toothbrush	Ra μm Mean ± SD (95% CI)	Rq μm Mean ± SD (95% CI)	Sa μm Mean ± SD (95% CI)
**ADA reference manual toothbrush**	17.43 ± 0.89 (16.01–18.82)^a^	22.56 ± 0.91 (21.23–24.09)^a^	17.91 ± 0.86 (17.19–18.63)^a^
**Curaprox Hydrosonic Pro**	14.00 ± 0.74 (13.09–14.95)^b^	17.65 ± 1.16 (16.52–19.88)^b^	14.45 ± 0.69 (13.87–15.03)^b^
**Curaprox Hydrosonic Black is White**	14.56 ± 0.76 (13.58–15.58)^b^	18.59 ± 1.05 (16.64–19.94)^b^	14.96 ± 0.79 (14.30–15.62)^b^

Within each column, values with the same superscript letter are not significantly different (p > 0.05), whereas values with different letters are significantly different (p < 0.001; one-way ANOVA with the Bonferroni post hoc correction).

The complete dataset is available in the Supporting Information (S1 and S2 Files).

## Discussion

Based on the results of this study, H01—assuming that there was no statistically significant difference in dentin abrasion caused by the charcoal-coated sonic toothbrush, the uncoated sonic toothbrush, and the ADA reference manual toothbrush—was partially rejected. Compared with the ADA reference manual toothbrush, both the sonic toothbrushes caused significantly greater dentin abrasion, but no significant difference in dentin abrasion was found between the two sonic toothbrushes; these results indicate that the filament coating did not influence abrasion under the conditions tested.

H02—assuming that there was no statistically significant difference in surface roughness caused by the tested toothbrushes—was also partially rejected. Both types of sonic toothbrushes produced dentin surfaces with significantly less roughness than the ADA reference manual toothbrush did, while no significant difference in surface roughness was observed between the two types of sonic toothbrushes.

While human dentin remains the gold standard for clinical relevance, bovine dentin provides a scientifically valid, ethical, and highly practical alternative for in vitro dentin abrasion studies. Its structural and mechanical similarity to human dentin, combined with greater uniformity, standardized preparation, and ease of accessibility, make bovine dentin particularly suitable for controlled and reproducible laboratory research [24,25]. The use of bovine dentin is also in accordance with ISO 11609 requirements, which specify substrates free of preexisting wear or defects [15]. In this study, only sound dentin confirmed by hardness testing was included to exclude the effects of preexisting demineralization or caries [26].

Three toothbrushes were tested to compare the effects of the filament coating and brushing mode on dentin wear; these toothbrushes included ADA reference manual toothbrush, the uncoated sonic toothbrush (Curaprox Hydrosonic Pro), and the charcoal-coated sonic toothbrush (Curaprox Hydrosonic Black is White). The sonic models allowed direct comparison of the effects of the charcoal coating, while the ADA reference served as a standardized control in line with ISO 11609 and previous in vitro studies [5,17,27]. In addition, manual toothbrushes remain more widely used than electric toothbrushes in Germany, as reported in recent market analyses [28].

To our knowledge, this study is among the first to quantify dentin abrasion and surface roughness associated with charcoal-coated toothbrush filaments using a standardized in vitro protocol. By testing two otherwise identical sonic toothbrush models that differed only in terms of filament coating, the specific influence of charcoal application on dentin wear could be isolated. This design enables a direct assessment of coating effects without confounding differences in brush head geometry, filament stiffness, or tuft arrangement.

A toothpaste slurry was used instead of water, as water alone produces negligible abrasive effects [29] and does not reflect realistic use. This approach is consistent with the intended application of the tested brushes, particularly charcoal-coated models, which are typically paired with whitening toothpaste. Using a standardized slurry ensured reproducibility and comparability with other in vitro abrasion studies.

Key strengths include strict adherence to ISO-based methodology, standardized brushing force and motion, blinded sample allocation, and the use of high-precision noncontact optical profilometry to achieve reproducible measurements of dentin loss and surface roughness. The sample size, which was determined from the preliminary data of this study [17] and supported by findings from Greuling et al. [11], exceeded the calculated minimum to account for potential dropouts and to enhance the robustness and reproducibility of the results.

The results indicate that toothbrush type, rather than charcoal filament coating, was the primary determinant of dentin wear and postbrushing surface roughness. Both sonic models caused greater abrasion than the ADA reference manual toothbrush, which is consistent with previous reports of greater dentin loss from activated electric toothbrushes under standardized load and cycle conditions [17,30]. Differences in filament geometry and density may contribute; Dyer et al. [31] demonstrated that thinner, more densely packed filaments retain more toothpaste, are more readily flexible, and produce greater surface contact, increasing abrasivity. In addition, the greater bristle travel distance of sonic toothbrushes than of manual brushes, even under identical brushing forces, may further explain their greater abrasivity. As reported by Bizhang et al. [17], the activated oscillation of sonic brushes eliminates the need for extensive manual motion but results in substantially longer cumulative bristle displacement during the same brushing time, thereby increasing the mechanical work applied to the tooth surface. This increased movement distance, in accordance with the physical definition of work (W = F × s), likely contributed to the greater dentin loss observed with the sonic models in the present study.

No significant difference in dentin abrasion was observed between the charcoal-coated and uncoated sonic toothbrushes. This is consistent with studies on charcoal-containing toothpastes showing no additional abrasive effect attributable to charcoal when the RDA values are similar [12,13]. Although charcoal coatings are marketed for stain removal, our results suggest that they do not increase the wear of sound dentin under standardized conditions. However, the literature on charcoal-based abrasives remains controversial. Some investigations have reported that certain charcoal-containing toothpastes can lead to substantially greater dentin loss [32,33]. In a recent review, Brooks et al. [33] reported that only 28% of commercially available charcoal toothpastes were classified as having low abrasiveness, highlighting the variability among products and the need for case-specific evaluation, especially regarding their abrasivity.

An inverse relationship was observed between dentin loss and surface roughness: toothbrushes causing greater abrasion tended to result in less roughness. This is consistent with profilometric principles, as the removal of surface peaks by abrasive action reduces the measured roughness values. Similar results have been reported in previous studies, suggesting that surface smoothing may be an inherent consequence of material removal rather than a direct indicator of gentleness [10,34].

From a clinical perspective, while sonic toothbrushes—regardless of the use of a filament coating—may produce dentin surfaces with less roughness, this benefit may come at the cost of greater hard tissue loss. Practitioners should advise patients with exposed dentin to balance cleaning efficacy against the risk of increased abrasion and tailor recommendations to individual susceptibility.

## Conclusion

Within the limitations of this in vitro study, toothbrush type—rather than a charcoal filament coating—was the primary factor that affected dentin wear and surface texture. These findings emphasize the importance of considering toothbrush design in patients with exposed dentin and provide a basis for further in vivo confirmation under real-life brushing conditions.

## Supporting information

S1 File Secondary profilometry dataset of all abrasion measurements and surface roughness used in the present study. (XLSX)

S2 File Raw data file. (XLSX)
